# Association of Social Risk Domains With Poor Cardiovascular Risk Factor Control in US Adults With Diabetes, From 2006 to 2016

**DOI:** 10.1001/jamanetworkopen.2022.30853

**Published:** 2022-09-09

**Authors:** Timothy Corwin, Mukoso N. Ozieh, Emma Garacci, Rebekah J. Walker, Leonard E. Egede

**Affiliations:** 1 Medical College of Wisconsin School of Medicine, Milwaukee; 2 Center for Advancing Population Science, Medical College of Wisconsin, Milwaukee; 3 Division of Nephrology, Department of Medicine, Medical College of Wisconsin, Milwaukee; 4 Division of Nephrology, Clement J. Zablocki VA Medical Center, Milwaukee, Wisconsin; 5 Division of General Internal Medicine, Department of Medicine, Medical College of Wisconsin, Milwaukee

## Abstract

**Importance:**

Few studies have examined the association between social risk factors and poor control of cardiovascular disease (CVD) risk factors.

**Objective:**

To examine the sequential association between social risk domains and CVD risk control over time in older adults with diabetes.

**Design, Setting, and Participants:**

This cohort study analyzed core interviews from 4877 US adults with diabetes who were participating in the Health and Retirement Study, a national longitudinal survey of US adults. Participants were older than 50 years, completed the social risk domain questions, and had data on CVD risk factor measures from January 2006 to December 2016. Data were analyzed from June to July 2022.

**Exposures:**

Five social risk domains were created: (1) economic stability, (2) neighborhood or built environment, (3) education access, (4) health care access, and (5) social or community context.

**Main Outcomes and Measures:**

The 4 primary outcomes were (1) poor glycemic control (hemoglobin A_1c_ [HbA_1c_] level ≥8.0%), (2) poor blood pressure (BP) control (systolic BP≥140 mm Hg and diastolic BP ≥90 mm Hg), (3) poor cholesterol control (total cholesterol/high-density lipoprotein ratio ≥5), and (4) a composite of poor CVD risk control (≥2 poorly controlled glucose level, BP, or cholesterol level).

**Results:**

Among this cohort of 4877 older adults with diabetes (mean [SD] age, 68.6 [9.8] years; 2715 women [55.7%]), 890 participants (18.3%) had an HbA_1c_ level of 8% or higher, 774 (15.9%) had systolic BP of 140 mm Hg or higher and diastolic BP of 90 mm Hg or higher, 962 (19.7%) had total cholesterol/high-density lipoprotein ratio of 5 or higher, and 437 (9.0%) had at least 2 poorly controlled CVD risk factors. Neighborhood or built environment (ie, adverse social support) was independently associated with poor glycemic control (odds ratio [OR], 1.31; 95% CI, 1.06-1.63), whereas economic stability (ie, medication cost–related nonadherence) (OR, 1.40; 95% CI, 1.04-1.87) and health care access (ie, lack of health insurance) (OR, 1.58; 95% CI, 1.20-2.09) were independently associated with poor BP control after full adjustment. Education access (ie, lack of education) (OR, 1.24; 95% CI, 1.01-1.52) and health care access (ie, lack of health insurance) (OR, 1.31; 95% CI, 1.02-1.68) were independently associated with poor cholesterol control. Health care access (ie, lack of health insurance) was the only social risk domain that was independently associated with having at least 2 poorly controlled CVD risk factors (OR, 1.72; 95% CI, 1.26-2.37).

**Conclusions and Relevance:**

Results of this study suggest that certain social risk domains are associated with control of CVD risk factors over time. Interventions targeting domains, such as neighborhood or built environment, economic stability, and education access, may be beneficial to controlling CVD risk factors in older adults with diabetes.

## Key Points

**Question:**

What is the longitudinal association between social risk domains and poor control of cardiovascular disease risk factors in older adults with diabetes?

**Findings:**

In this cohort study of 4877 older adults with diabetes, the social risk domain of neighborhood or built environment (ie, adverse social support) was independently associated with poor glycemic control, economic stability (ie, medication cost–related nonadherence) and health care access (ie, lack of health insurance) were independently associated with poor blood pressure control, and education access (ie, lack of education) and health care access (ie, lack of health insurance) were independently associated with poor cholesterol control.

**Meaning:**

Findings of this study suggest that some social risk domains may be associated with cardiovascular disease risk factors in older adults with diabetes.

## Teaser

This cohort study assesses the management of glucose level, blood pressure, and cholesterol level within the context of social determinants of health among older adults with diabetes in the US.

## Introduction

In the US, 37.3 million people have diabetes, and 90% to 95% of them have type 2 diabetes.^1,2^ Diabetes is highly prevalent in older US adults, with an estimated prevalence of 29% in adults 65 years or older and 24% in younger adults.^1^ Furthermore, diabetes is the seventh leading cause of death in the US and a common cause of decreased life expectancy.^2,3^ Mortality from all causes is highest in people with diabetes, but cardiovascular disease (CVD) and CVD risk factors play a major role in mortality in diabetes.^3,4^

Control of CVD risk factors is critical to reducing morbidity and mortality in diabetes. Traditional CVD risk factors include high blood pressure (BP), high blood glucose level, and high cholesterol level.^1^ According to the Centers for Disease Control and Prevention, control of traditional CVD risk factors is suboptimal among individuals with a diabetes diagnosis, of whom 69% had poorly controlled BP (defined as systolic BP [SBP] ≥140 mm Hg or diastolic BP [DBP] ≥90 mm Hg), 49% had poorly controlled blood glucose level (defined as hemoglobin A_1c_ [HbA_1c_] level ≥7% [to convert to the proportion of total hemoglobin, multiply by 0.01]), and 44% had poorly controlled cholesterol level (defined as non–high-density lipoprotein [HDL] level ≥130 mg/dL [to convert to millimoles per liter, multiply by 0.0259]).^1^ Evidence suggests that controlling these traditional risk factors in combination with lifestyle behavioral changes saves lives.^1,2,4^ However, evidence also suggests that controlling traditional risk factors alone is inadequate for reducing poor outcomes in diabetes, including CVD and mortality.^5^

Social determinants of health (SDOH) are emerging as key factors associated with poor risk factor control, morbidity, and mortality.^5,6^ Although there is no consensus regarding factors that define SDOH^5^ or consistent terminology for SDOH,^7^ certain domains are consistent across existing SDOH frameworks: economic stability, including low income and employment adversity; neighborhood or built environment, including food insecurity and poor environmental conditions; education, including lack of access to education; health and health care, including lack of access to health care and health insurance; and community and social context, including perceived discrimination and stress.^5^ These domains are recognized as adverse social conditions and social risk factors.^7,8,9,10,11,12,13,14,15,16,17^ There is limited evidence on the implications of these domains for controlling CVD risk factors over time.

In this study, we used a nationally representative cohort of older adults with diabetes to examine the sequential association between social risk domains and CVD risk control over time. We hypothesized that individual social risk domains are associated with poor control of CVD risk factors over time, even after adjusting for relevant covariates.

## Methods

Institutional review board approval and informed consent requirement were waived for this retrospective cohort study by the Medical College of Wisconsin because the study used the publicly available Health and Retirement Study (HRS) database, which contains deidentified data. We followed the Strengthening the Reporting of Observational Studies in Epidemiology (STROBE) reporting guideline.

### Data Source and Study Population

The HRS is a national longitudinal survey of US adults older than 50 years and their spouses.^18^ The main goal of the HRS is to examine the ways in which the changing health of older adults interacts with the social, economic, and psychological aspects of their lives and retirement decisions. Since the first wave of the HRS in 1992, respondents have been interviewed every 2 years regarding their financial, health, and family status. New cohorts of respondents aged 51 to 56 years and their spouses are added to the HRS every 6 years, maintaining its status as nationally representative of households with members older than 50 years. Biennial interviews are conducted, and data through 2016 have been released.^19^ The enhanced face-to-face interview includes a set of physical performance measures, a collection of biomarkers, and a Leave-Behind Questionnaire on psychosocial topics.^20^ A random half of households were preselected for the enhanced face-to-face interview in 2006, and the other half of the sample was selected for 2008. Since then, every household repeats the enhanced face-to-face interview for every other wave.

We analyzed the core interviews from 2006 to 2016.^21^ The cohort for the present study included individuals who were older than 50 years, participated in the biomarker project of the HRS, completed the Leave-Behind Questionnaire, had complete data for the social risk domain questions, and had a physician’s diagnosis of diabetes or an HbA_1c_ level of 6.5% or higher in the biomarker data. These participants were interviewed a maximum of 3 times during the study period, with a group of individuals interviewed in 2006, 2010, and 2014 and another group interviewed in 2008, 2012, and 2016. As a result, each participant was in the HRS for a duration of 8 years.

### Primary Outcomes

Four primary outcomes were established to capture control of cardiovascular risk factors: (1) poor glycemic control, defined as an HbA_1c_ level of 8.0% or higher; (2) poor BP control, defined as SBP of 140 mm Hg or higher and DBP of 90 mm Hg or higher; (3) poor cholesterol control, defined as total cholesterol/HDL (TC/HDL) ratio of 5 or higher; and (4) a composite measure of poor CVD risk factor control, defined as 2 or more poorly controlled outcomes (eg, poor glycemic and poor BP control, or poor BP and poor cholesterol control).

Blood pressure was measured during the enhanced face-to-face interview, whereas the HbA_1c_ level and TC/HDL ratio were assayed from dried blood spot samples collected during the interview. The HRS constructed and released a National Health and Nutrition Examination Survey (NHANES)–equivalent assay value for each assay because the resulting biomarker values that were based on dried blood spot samples varied across assays and laboratories. In addition, these samples may be different from the conventional whole blood assays; hence, NHANES recommends the equivalent assay values for analytic use.^21^ We used the NHANES-equivalent values for analysis.

### Primary Independent Variables

Five social risk domains were created according to the modified Kaiser Family Foundation conceptual SDOH framework.^22^ The domains were (1) economic stability, (2) neighborhood or built environment, (3) education access, (4) health care access, and (5) social or community context. Consistent with previous work,^23^ we used the questions asked at the baseline interview to map to each of these domains, and then we created binary indicators for questions in each domain.

The 5 variables of the economic stability domain included medication cost–related nonadherence, based on not using prescribed medications because of their high cost; difficulty paying bills, based on a yes response to this question; financial hardship, based on several questions assessing financial stability; lowest quartile of income or assets, based on a yes response to this question; and employment adversity, based on being unemployed. Four variables of the neighborhood or built environment domain included food insecurity, based on a yes response to this question; lack of social cohesion (score range, 1-7), based on a score of 4 or lower on this question; neighborhood physical disorder, based on a score of 4 or higher on these questions; and adverse social support, based on a score lower than 3 on these questions.

For the education access domain, the indicator variable was based on an education level lower than a high school diploma. For the health care access domain, the indicator variable was based on lack of health insurance. For the social or community context domain, the 2 indicators were based on depression and perceived discrimination. Depression was defined as a score of 4 or higher on the 8-item Center for Epidemiological Studies-Depression scale (score range: 0-24, with higher scores indicating higher frequency of depressive symptoms), and perceived discrimination was based on a score of 2 or higher on the Everyday Discrimination Scale (score range: 0-5, with higher scores indicating more frequent experiences of everyday discrimination).

### Covariates

Demographic factors included sex, age, race and ethnicity (which were self-identified by participants and categorized as non-Hispanic Black; non-Hispanic White; and other minority groups, such as American Indian and Alaska Native, Asian, Hispanic, and Native Hawaiian or Pacific Islander), and marital status (yes or no response to the question about married status or living with a partner). Comorbidities included high BP, cancer, lung disease, heart disease (eg, heart attack, angina, congestive heart failure), stroke, emotional or psychiatric problems, arthritis, and dementia. Individuals who indicated being diagnosed with any of these conditions by a health care professional were coded as having the comorbidity. Each comorbidity was added separately into the models.

### Statistical Analysis

Frequency counts of demographic factors, social risk domains, covariates, and outcome measures were assessed. Mixed-effects logistic regression was used to model the association over time between the 5 social risk domains and the 4 primary outcomes of CVD risk factor control, adjusting for covariates and accounting for repeated measures and nonindependence of measures over time for each individual. The model included a random effect for each individual. Unadjusted mixed-effects logistic regression was used to assess the independent association between each social risk domain and each primary outcome of CVD risk factor control. Four sequential mixed-effects logistic regressions (models 1-4) were performed to assess the independent association between social risk domains and each primary outcome of CVD risk factor control. For the adjusted model (model 5), the primary outcome was each of the 4 CVD risk factor control outcomes; the primary independent variables were the 5 social risk domains; and the covariates were sex, age, race and ethnicity, marital status, and comorbidities. Variables were entered in blocks.

Model 1 included economic stability variables and neighborhood or built environment variables. Model 2 included economic stability variables, neighborhood or built environment variables, and education access variables. Model 3 included economic stability variables, neighborhood or built environment variables, education access variables, and health care access variables. Model 4 included economic stability variables, neighborhood or built environment variables, education access variables, health care access variables, and social or community context variables. Model 5 included economic stability variables, neighborhood or built environment variables, education access variables, health care access variables, social or community context variables, and covariates.

Statistical analyses were performed with Stata, version 16.0 (StataCorp), using survey design methods to account for the complex sampling design and nationally representative weights. All tests were 2-sided, and *P* < .05 was considered to be statistically significant. Data were analyzed from June to July 2022.

## Results

Of the 18 777 older individuals who participated in the HRS biomarker project and completed the Leave-Behind Questionnaire, 18 114 completed the social risk domain questions during the study period, and 16 701 had data on the CVD risk factor measures. Among this population, 4877 had diabetes and were included in all analyses.

Table 1 shows the baseline demographic characteristics, comorbidities, responses to questions within each social risk domain, and outcomes. The cohort had a mean (SD) age of 68.6 (9.8) years; had 2715 women (55.7%) and 2162 men (44.3%); and was predominantly composed of non-Hispanic White participants (2869 [58.9%]) who were married or living with a partner (3123 [64.0%]). During the study period, 890 participants (18.3%) had HbA_1c_ level of 8% or higher, 774 (15.9%) had SBP of 140 mm Hg or higher and DBP of 90 mm Hg or higher, 962 (19.7%) had TC/HDL ratio of 5 or higher, and 437 (9.0%) had at least 2 poorly controlled CVD risk factors.

**Table 1.** Baseline Characteristics of Older Adults With Diabetes in the Health and Retirement Study, From January 2006 to December 2016

Characteristic	No. (%)
Adults with diabetes	4877
Age, y	
Continuous, mean (SD)	68.6 (9.8)
Categorical	
50-59	1329 (27.3)
60-74	2660 (54.5)
≥75	1566 (32.1)
Sex	
Male	2162 (44.3)
Female	2715 (55.7)
Race and ethnicity^a^	
Non-Hispanic Black	1099 (22.5)
Non-Hispanic White	2869 (58.9)
Other	907 (18.6)
Married or living with a partner	
Yes	3123 (64.0)
No	1945 (39.9)
High blood pressure	
Yes	3886 (79.7)
No	1164 (23.9)
Cancer	
Yes	870 (17.9)
No	4117 (84.5)
Lung disease	
Yes	691 (14.2)
No	4293 (88.1)
Heart disease	
Yes	1755 (36.0)
No	3365 (69.0)
Stroke	
Yes	506 (10.4)
No	4458 (91.4)
Emotional or psychiatric problems	
Yes	1119 (23.0)
No	3880 (79.6)
Arthritis	
Yes	3326 (68.2)
No	1756 (36.0)
Dementia	
Yes	4830 (99.0)
No	93 (1.9)
Economic stability	
Medication cost–related nonadherence	
Yes	918 (18.8)
No	4294 (88.1)
Difficulty paying bills	
Yes	2142 (43.9)
No	3224 (66.1)
Financial hardship	
Yes	2422 (49.7)
No	2960 (60.7)
Lowest-quartile income or assets	
Yes	1612 (33.1)
No	3562 (73.0)
Employment adversity	
Yes	984 (20.2)
No	4126 (84.6)
Neighborhood or built environment	
Food insecurity	
Yes	401 (8.2)
No	4604 (94.4)
Neighborhood physical disorder	
Yes	1411 (28.9)
No	3948 (81.0)
Lack of neighborhood social cohesion	
Yes	1423 (29.2)
No	3924 (80.5)
Adverse social support	
Yes	1678 (34.4)
No	3637 (74.6)
Education access	
Lack of education	
Yes	1102 (22.6)
No	3775 (77.4)
Health care access	
Health insurance	
Yes	4441 (91.1)
No	642 (13.2)
Social or community context	
Depression	
Yes	1059 (21.7)
No	4136 (84.8)
Perceived everyday discrimination	
Yes	1573 (32.3)
No	3760 (77.1)
Outcomes	
Blood hemoglobin (HbA_1c_)	
<8.0%	4354 (89.3)
≥8.0%	890 (18.3)
Blood pressure, mm Hg	
SBP <140 or DBP <90	4434 (90.9)
SBP ≥140 and DBP ≥90	774 (15.9)
Cholesterol	
TC/HDL ratio <5	4350 (89.2)
TC/HDL ratio ≥5	962 (19.7)
CVD risk at least 2 out of control	
Yes	437 (9.0)
No	4645 (95.2)

Abbreviations: CVD, cardiovascular disease; DBP, diastolic blood pressure; HbA_1c_, hemoglobin A_1c_; SBP, systolic blood pressure; TC/HDL, total cholesterol/high-density lipoprotein.

^a^ Race and ethnicity were self-identified by participants. Other race and ethnicity groups included American Indian and Alaska Native, Asian, Hispanic, and Native Hawaiian or Pacific Islander.

Results of univariate analyses using mixed-effects logistic regression models to examine the association over time between social risk domains and clinical outcomes are shown in eTable in the Supplement. All 5 social risk domains were associated with a poorly controlled HbA_1c_ level and having at least 2 poorly controlled CVD risk factors. For example, the lowest-quartile income or assets in the economic stability domain, food insecurity in the neighborhood or built environment domain, lack of education in the education access domain, lack of health insurance in the health care access domain, and perceived everyday discrimination in the social or community context domain were all associated with poorly controlled HbA_1c_ level. Economic stability (medication cost–related nonadherence), neighborhood or built environment (food insecurity), education access (lack of education), and health care access (lack of health insurance) domains were all associated with poorly controlled BP over time. However, all social risk domains (including adverse social support in the neighborhood or built environment domain, lack of education in the education access domain, lack of health insurance in the health care access domain, and perceived everyday discrimination in the social or community context domain) except economic stability were associated with poor cholesterol control.

Table 2 shows the association over time between all 5 social risk domains and glycemic control outcome. Moreover, non-Hispanic Black older adults were more likely to have poor glycemic control compared with non-Hispanic White older adults (odds ratio [OR], 1.97; 95% CI, 1.51-2.56). In model 5, adjusting for all social risk domain variables and covariates, only the association of adverse social support variable (OR, 1.31; 95% CI, 1.06-1.63) in the neighborhood or built environment domain maintained statistical significance.

**Table 2.** Adjusted Mixed-Effects Logistic Regression With Social Risk Domains in Added Blocks for Glycemic Control Outcome^a^

	HbA_1c_ ≥8.0%, OR (95% CI)				
	Model 1^b^	Model 2^c^	Model 3^d^	Model 4^e^	Model 5^f^
Economic stability					
Medication cost–related nonadherence	1.13 (0.83-1.54)	1.14 (0.84-1.56)	1.10 (0.81-1.50)	1.08 (0.79-1.47)	1.10 (0.81-1.50)
Difficulty paying bills	1.24 (0.79-1.94)	1.26 (0.81-1.97)	1.22 (0.78-1.90)	1.20 (0.77-1.88)	1.25 (0.80-1.93)
Financial hardship	1.20 (0.74-1.95)	1.17 (0.72-1.90)	1.19 (0.73-1.92)	1.19 (0.74-1.93)	1.03 (0.64-1.66)
Lowest-quartile income or assets	1.26 (1.00-1.59)^a^	1.20 (0.95-1.51)	1.18 (0.94-1.49)	1.17 (0.93-1.48)	0.99 (0.78-1.27)
Employment adversity	1.17 (0.90-1.52)	1.16 (0.89-1.51)	1.15 (0.89-1.50)	1.12 (0.86-1.46)	0.99 (0.75-1.29)
Neighborhood or built environment					
Food insecurity	1.22 (0.83-1.78)	1.22 (0.83-1.78)	1.22 (0.84-1.79)	1.20 (0.82-1.76)	1.13 (0.78-1.64)
Neighborhood physical disorder	1.09 (0.84-1.41)	1.07 (0.82-1.40)	1.08 (0.83-1.40)	1.08 (0.83-1.39)	1.01 (0.78-1.31)
Lack of neighborhood social cohesion	1.36 (1.04-1.77)^a^	1.34 (1.03-1.75)^a^	1.33 (1.02-1.73)^a^	1.33 (1.02-1.72)^a^	1.24 (0.96-1.60)
Adverse social support	1.42 (1.15-1.76)^a^	1.43 (1.16-1.77)^a^	1.42 (1.15-1.75)^a^	1.38 (1.11-1.72)^a^	1.31 (1.06-1.63)^a^
Education access					
Lack of education	NA	1.32 (1.03-1.69)^a^	1.31 (1.02-1.68)^a^	1.30 (1.01-1.67)^a^	1.21 (0.94-1.56)
Health care access					
Lack of health insurance	NA	NA	1.74 (1.35-2.33)^a^	1.75 (1.31-2.33)^a^	1.30 (0.96-1.75)
Social or community context					
Depression	NA	NA	NA	1.18 (0.92-1.53)	1.20 (0.92-1.55)
Perceived everyday discrimination	NA	NA	NA	1.02 (0.81-1.27)	0.96 (0.77-1.20)
Covariates					
High blood pressure	NA	NA	NA	NA	0.79 (0.62-1.00)
Cancer	NA	NA	NA	NA	0.78 (0.59-1.04)
Lung disease	NA	NA	NA	NA	0.82 (0.60-1.12)
Heart disease	NA	NA	NA	NA	1.24 (0.99-1.55)
Stroke	NA	NA	NA	NA	1.06 (0.75-1.50)
Emotional or psychiatric problems	NA	NA	NA	NA	1.04 (0.80-1.34)
Arthritis	NA	NA	NA	NA	0.95 (0.77-1.19)
Dementia	NA	NA	NA	NA	1.56 (0.75-3.26)
Age, y					
60-74	NA	NA	NA	NA	0.65 (0.51-0.82)^a^
≥75	NA	NA	NA	NA	0.49 (0.36-0.67)^a^
Race and ethnicity^g^					
Non-Hispanic Black	NA	NA	NA	NA	1.97 (1.51-2.56)^a^
Other	NA	NA	NA	NA	2.18 (1.63-2.90)^a^
Married or living with a partner	NA	NA	NA	NA	0.85 (0.68-1.07)
Sex					
Male	NA	NA	NA	NA	1 [Reference]
Female	NA	NA	NA	NA	0.74 (0.59-0.92)^a^

Abbreviations: HbA_1c_, hemoglobin A_1c_; NA, not applicable; OR, odds ratio.

^a^ *P* < .05.

^b^ Model 1 included economic stability variables and neighborhood or built environment variables.

^c^ Model 2 included economic stability variables, neighborhood or built environment variables, and education access variables.

^d^ Model 3 included economic stability variables, neighborhood or built environment variables, education access variables, and health care access variables.

^e^ Model 4 included economic stability variables, neighborhood or built environment variables, education access variables, health care access variables, and social or community context variables.

^f^ Model 5 included economic stability variables, neighborhood or built environment variables, education access variables, health care access variables, social or community context variables, and covariates.

^g^ Race and ethnicity were self-identified by participants. Other race and ethnicity groups included American Indian and Alaska Native, Asian, Hispanic, and Native Hawaiian or Pacific Islander.

Table 3 shows the association over time between all 5 social risk domains and BP control outcome. Medication cost–related nonadherence in the economic stability domain (OR, 1.40; 95% CI, 1.04-1.87) and lack of health insurance in the health care access domain (OR, 1.58; 95% CI, 1.20-2.09) were independently associated with poorly controlled BP after full adjustment. However, findings in the neighborhood or built environment, education access, and social or community context domains were not statistically significant.

**Table 3.** Adjusted Mixed-Effects Logistic Regression With Social Risk Domains in Added Blocks for BP Control Outcome^a^

	SBP≥140 and BP≥90 mm Hg, OR (95% CI)				
	Model 1^b^	Model 2^c^	Model 3^d^	Model 4^e^	Model 5^f^
Economic stability					
Medication cost–related nonadherence	1.46 (1.09-1.94)^a^	1.48 (1.11-1.97)^a^	1.43 (1.07-1.91)^a^	1.42 (1.06-1.89)^a^	1.40 (1.04-1.87)^a^
Difficulty paying bills	1.08 (0.72-1.61)	1.09 (0.73-1.63)	1.05 (0.70-1.58)	1.04 (0.70-1.56)	1.10 (0.73-1.65)
Financial hardship	0.97 (0.63-1.50)	0.96 (0.62-1.48)	0.97 (0.62-1.50)	0.97 (0.63-1.51)	0.91 (0.59-1.42)
Lowest-quartile income or assets	1.38 (1.12-1.70)^a^	1.33 (1.08-1.65)^a^	1.32 (1.06-1.63)^a^	1.32 (1.06-1.63)^a^	1.16 (0.92-1.46)
Employment adversity	1.02 (0.79-1.30)	1.01 (0.79-1.29)	0.99 (0.78-1.27)	0.99 (0.77-1.26)	0.92 (0.71-1.19)
Neighborhood or built environment					
Food insecurity	1.19 (0.84-1.69)	1.19 (0.84-1.69)	1.20 (0.85-1.70)	1.19 (0.84-1.69)	1.14 (0.80-1.62)
Neighborhood physical disorder	1.15 (0.90-1.47)	1.14 (0.89-1.46)	1.14 (0.89-1.47)	1.14 (0.89-1.46)	1.10 (0.85-1.41)
Lack of neighborhood social cohesion	0.88 (0.68-1.13)	0.87 (0.68-1.13)	0.86 (0.67-1.11)	0.86 (0.66-1.11)	0.83 (0.64-1.07)
Adverse social support	0.95 (0.78-1.16)	0.95 (0.78-1.16)	0.95 (0.78-1.16)	0.92 (0.75-1.14)	0.91 (0.74-1.13)
Education access					
Lack of education	NA	1.19 (0.96-1.49)	1.18 (0.95-1.48)	1.18 (0.95-1.48)	1.25 (0.99-1.58)
Health care access					
Lack of health insurance	NA	NA	1.72 (1.32-2.24)^a^	1.73 (1.32-2.25)^a^	1.58 (1.20-2.09)^a^
Social or community context					
Depression	NA	NA	NA	1.04 (0.81-1.32)	1.08 (0.84-1.39)
Perceived everyday discrimination	NA	NA	NA	1.10 (0.89-1.35)	1.08 (0.87-1.33)
Covariates					
High BP	NA	NA	NA	NA	1.83 (1.43-2.34)^a^
Cancer	NA	NA	NA	NA	0.59 (0.45-0.78)^a^
Lung disease	NA	NA	NA	NA	0.90 (0.68-1.20)
Heart disease	NA	NA	NA	NA	0.81 (0.66-1.00)
Stroke	NA	NA	NA	NA	1.02 (0.74-1.41)
Emotional or psychiatric problems	NA	NA	NA	NA	0.82 (0.65-1.05)
Arthritis	NA	NA	NA	NA	0.82 (0.67-0.99)^a^
Dementia	NA	NA	NA	NA	1.17 (0.55-2.48)
Age, y					
60-74	NA	NA	NA	NA	0.92 (0.73-1.16)
≥75	NA	NA	NA	NA	0.67 (0.50-0.91)^a^
Race and ethnicity^g^					
Non-Hispanic Black	NA	NA	NA	NA	1.38 (1.09-1.75)^a^
Other	NA	NA	NA	NA	0.90 (0.69-1.19)
Married or living with a partner	NA	NA	NA	NA	0.76 (0.61-0.93)^a^
Sex					
Male	NA	NA	NA	NA	1 [Reference]
Female	NA	NA	NA	NA	0.95 (0.78-1.16)

Abbreviations: BP, blood pressure; NA, not applicable; OR, odds ratio; SBP, systolic blood pressure.

^a^ *P* < .05.

^b^ Model 1 included economic stability variables and neighborhood or built environment variables.

^c^ Model 2 included economic stability variables, neighborhood or built environment variables, and education access variables.

^d^ Model 3 included economic stability variables, neighborhood or built environment variables, education access variables, and health care access variables.

^e^ Model 4 included economic stability variables, neighborhood or built environment variables, education access variables, health care access variables, and social or community context variables.

^f^ Model 5 included economic stability variables, neighborhood or built environment variables, education access variables, health care access variables, social or community context variables, and covariates.

^g^ Race and ethnicity were self-identified by participants. Other race and ethnicity groups included American Indian and Alaska Native, Asian, Hispanic, and Native Hawaiian or Pacific Islander.

Table 4 provides the association over time between all 5 social risk domains and cholesterol control outcome. Lack of education in the education access domain (OR, 1.24; 95% CI, 1.01-1.52) and lack of health insurance in the health care access domain (OR, 1.31; 95% CI, 1.02-1.68) had an independent association with poor cholesterol control over time in the fully adjusted model. However, no association was observed for the variables in the economic stability, neighborhood or built environment, and social or community context domains.

**Table 4.** Adjusted Mixed-Effects Logistic Regression With Social Risk Domains in Added Blocks for Cholesterol Control Outcome^a^

	TC/HDL ratio ≥5, OR (95% CI)				
	Model 1^b^	Model 2^c^	Model 3^d^	Model 4^e^	Model 5^f^
Economic stability					
Medication cost–related nonadherence	1.22 (0.94-1.59)	1.24 (0.95-1.61)	1.21 (0.93-1.57)	1.19 (0.91-1.55)	1.20 (0.92-1.56)
Difficulty paying bills	1.22 (0.83-1.78)	1.23 (0.84-1.79)	1.20 (0.82-1.75)	1.18 (0.81-1.73)	1.19 (0.81-1.74)
Financial hardship	0.85 (0.57-1.28)	0.84 (0.56-1.26)	0.85 (0.56-1.27)	0.85 (0.57-1.28)	0.85 (0.56-1.28)
Lowest-quartile income or assets	1.15 (0.95-1.39)	1.11 (0.91-1.34)	1.10 (0.91-1.33)	1.09 (0.90-1.32)	1.22 (0.99-1.49)
Employment adversity	1.08 (0.87-1.34)	1.07 (0.86-1.33)	1.07 (0.86-1.32)	1.05 (0.85-1.31)	1.03 (0.82-1.29)
Neighborhood or built environment					
Food insecurity	0.82 (0.59-1.15)	0.82 (0.59-1.15)	0.82 (0.59-1.15)	0.81 (0.58-1.13)	0.83 (0.59-1.16)
Neighborhood physical disorder	0.95 (0.76-1.18)	0.94 (0.75-1.17)	0.94 (0.75-1.17)	0.93 (0.75-1.16)	0.95 (0.76-1.19)
Lack of neighborhood social cohesion	1.02 (0.82-1.28)	1.02 (0.81-1.27)	1.01 (0.81-1.26)	1.01 (0.80-1.25)	1.01 (0.81-1.27)
Adverse social support	1.20 (1.01-1.42)^a^	1.20 (1.01-1.42)^a^	1.20 (1.01-1.42)^a^	1.15 (0.96-1.37)	1.08 (0.91-1.30)
Education access					
Lack of education	NA	1.20 (0.99-1.46)	1.20 (0.99-1.46)	1.20 (0.99-1.46)	1.24 (1.01-1.52)^a^
Health care access					
Lack of health insurance	NA	NA	1.45 (1.15-1.84)^a^	1.45 (1.15-1.84)^a^	1.31 (1.02-1.68)^a^
Social or community context					
Depression	NA	NA	NA	1.05 (0.85-1.29)	1.12 (0.90-1.40)
Perceived everyday discrimination	NA	NA	NA	1.16 (0.97-1.38)	1.11 (0.93-1.33)
Covariates					
High blood pressure	NA	NA	NA	NA	0.97 (0.80-1.17)
Cancer	NA	NA	NA	NA	0.83 (0.66-1.03)
Lung disease	NA	NA	NA	NA	1.12 (0.88-1.42)
Heart disease	NA	NA	NA	NA	0.95 (0.80-1.14)
Stroke	NA	NA	NA	NA	0.99 (0.75-1.31)
Emotional or psychiatric problems	NA	NA	NA	NA	0.88 (0.72-1.09)
Arthritis	NA	NA	NA	NA	1.14 (0.96-1.37)
Dementia	NA	NA	NA	NA	1.26 (0.68-2.35)
Age, y					
60-74	NA	NA	NA	NA	0.81 (0.66-0.99)^a^
≥75	NA	NA	NA	NA	0.72 (0.56-0.94)^a^
Race and ethnicity^g^					
Non-Hispanic Black	NA	NA	NA	NA	0.76 (0.61-0.95)^a^
Other	NA	NA	NA	NA	0.92 (0.73-1.16)
Married or living with a partner	NA	NA	NA	NA	1.12 (0.93-1.34)
Sex					
Male	NA	NA	NA	NA	1 [Reference]
Female	NA	NA	NA	NA	0.68 (0.57-0.81)^a^

Abbreviations: NA, not applicable; OR, odds ratio; TC/HDL, total cholesterol/high-density lipoprotein.

^a^ *P* < .05.

^b^ Model 1 included economic stability variables and neighborhood or built environment variables.

^c^ Model 2 included economic stability variables, neighborhood or built environment variables, and education access variables.

^d^ Model 3 included economic stability variables, neighborhood or built environment variables, education access variables, and health care access variables.

^e^ Model 4 included economic stability variables, neighborhood or built environment variables, education access variables, health care access variables, and social or community context variables.

^f^ Model 5 included economic stability variables, neighborhood or built environment variables, education access variables, health care access variables, social or community context variables, and covariates.

^g^ Race and ethnicity were self-identified by participants. Other race and ethnicity groups included American Indian and Alaska Native, Asian, Hispanic, and Native Hawaiian or Pacific Islander.

Table 5 shows the association over time between all 5 social risk domains and at least 2 poorly controlled CVD risk factors. Lack of health insurance in the health care access domain (OR, 1.72; 95% CI, 1.26-2.37) was the only variable that had an independent association with having at least 2 poorly controlled CVD risk factors.

**Table 5.** Adjusted Mixed-Effects Logistic Regression With Social Risk Domains in Blocks for 2 or More Poorly Controlled CVD Risk Outcomes^a^

	At least 2 poorly controlled CVD risks, OR (95% CI)				
	Model 1^b^	Model 2^c^	Model 3^d^	Model 4^e^	Model 5^f^
Economic stability					
Medication cost–related nonadherence	1.31 (0.93-1.85)	1.33 (0.94-1.87)	1.24 (0.88-1.75)	1.23 (0.87-1.74)	1.22 (0.86-1.73)
Difficulty paying bills	1.31 (0.79-2.18)	1.32 (0.79-2.20)	1.26 (0.75-2.10)	1.24 (0.74-2.08)	1.28 (0.77-2.14)
Financial hardship	1.08 (0.62-1.88)	1.07 (0.61-1.86)	1.08 (0.62-1.89)	1.08 (0.62-1.89)	0.98 (0.56-1.72)
Lowest-quartile income or assets	1.32 (1.02-1.70)^a^	1.27 (0.98-1.65)	1.25 (0.96-1.62)	1.25 (0.96-1.62)	1.19 (0.90-1.58)
Employment adversity	0.95 (0.70-1.28)	0.95 (0.70-1.28)	0.94 (0.70-1.27)	0.93 (0.69-1.26)	0.84 (0.61-1.15)
Neighborhood or built environment					
Food insecurity	0.80 (0.51-1.24)	0.80 (0.51-1.24)	0.81 (0.52-1.26)	0.80 (0.51-1.25)	0.78 (0.50-1.21)
Neighborhood physical disorder	1.06 (0.79-1.44)	1.05 (0.78-1.43)	1.07 (0.78-1.44)	1.05 (0.78-1.43)	1.04 (0.76-1.41)
Lack of neighborhood social cohesion	1.15 (0.85-1.55)	1.14 (0.85-1.55)	1.13 (0.83-1.53)	1.12 (0.83-1.52)	1.07 (0.79-1.45)
Adverse social support	1.22 (0.96-1.55)	1.22 (0.96-1.55)	1.21 (0.96-1.54)	1.17 (0.91-1.50)	1.11 (0.86-1.42)
Education access					
Lack of education	NA	1.18 (0.90-1.55)	1.18 (0.90-1.54)	1.18 (0.90-1.55)	1.27 (0.95-1.68)
Health care access					
Lack of health insurance	NA	NA	2.35 (1.74-3.18)^a^	2.35 (1.74-3.18)^a^	1.72 (1.26-2.37)^a^
Social or community context					
Depression	NA	NA	NA	0.99 (0.73-1.33)	1.11 (0.82-1.51)
Perceived everyday discrimination	NA	NA	NA	1.18 (0.91-1.51)	1.11 (0.86-1.44)
Covariates					
High blood pressure	NA	NA	NA	NA	1.16 (0.87-1.53)
Cancer	NA	NA	NA	NA	0.50 (0.34-0.74)^a^
Lung disease	NA	NA	NA	NA	0.95 (0.66-1.36)
Heart disease	NA	NA	NA	NA	1.09 (0.84-1.41)
Stroke	NA	NA	NA	NA	0.83 (0.54-1.28)
Emotional or psychiatric problems	NA	NA	NA	NA	0.80 (0.59-1.08)
Arthritis	NA	NA	NA	NA	0.88 (0.69-1.13)
Dementia	NA	NA	NA	NA	1.28 (0.50-3.31)
Age, y					
60-74	NA	NA	NA	NA	0.64 (0.49-0.85)^a^
≥75	NA	NA	NA	NA	0.41 (0.28-0.60)^a^
Race and ethnicity^g^					
Non-Hispanic Black	NA	NA	NA	NA	1.27 (0.95-1.70)
Other	NA	NA	NA	NA	1.15 (0.84-1.58)
Married or living with a partner	NA	NA	NA	NA	0.93 (0.72-1.20)
Sex					
Male	NA	NA	NA	NA	1 [Reference]
Female	NA	NA	NA	NA	0.74 (0.58-0.94)^a^

Abbreviations: CVD, cardiovascular disease; NA, not applicable; OR, odds ratio.

^a^ *P* < .05.

^b^ Model 1 included economic stability variables and neighborhood or built environment variables.

^c^ Model 2 included economic stability variables, neighborhood or built environment variables, and education access variables.

^d^ Model 3 included economic stability variables, neighborhood or built environment variables, education access variables, and health care access variables.

^e^ Model 4 included economic stability variables, neighborhood or built environment variables, education access variables, health care access variables, and social or community context variables.

^f^ Model 5 included economic stability variables, neighborhood or built environment variables, education access variables, health care access variables, social or community context variables, and covariates.

^g^ Race and ethnicity were self-identified by participants. Other race and ethnicity groups included American Indian and Alaska Native, Asian, Hispanic, and Native Hawaiian or Pacific Islander.

## Discussion

In this cohort study of older adults with diabetes in the US, we found that, in unadjusted models, social risk domains were associated with poor control of CVD risk factors. When adjusting for other domains and covariates, neighborhood or built environment (adverse social support) was independently associated with poor glycemic control, whereas economic stability (medication cost–related nonadherence) and health care access (lack of health insurance) were independently associated with poor BP control. Education access (lack of education) and health care access (lack of health insurance) were independently associated with poor cholesterol control. Health care access was the only social risk domain independently associated with having at least 2 poorly controlled CVD risk factors. In some fully adjusted models, race and ethnicity and sex were associated with poor control of CVD risk factors independent of social risk domains. This finding suggests that social risk factors compound the race and ethnicity–based and sex-based gaps in addressing CVD risk factors in this population.

Previous studies have examined the association of individual social risk domain variables, such as financial hardship, housing instability, chronic stress, or food insecurity, with health outcomes.^9,10,11,12,13,14,15,16,17,24^ However, to our knowledge, the present study was the first longitudinal analysis of the independent and sequential associations between social risk domains and poor CVD risk factor control among older adults with diabetes. Thus, this study adds to the literature by identifying that (1) the social risk domains of neighborhood or built environment, economic stability, and health care access have an association with poor CVD risk factor control over time; (2) lack of health insurance is associated with decreased control of CVD risk factors over time in older adults with diabetes; and (3) race and ethnicity and sex have implications for poor control of CVD risk factors over time independent of social risk factors. Specifically, non-Hispanic Black race and ethnicity or another minority racial and ethnic group was associated with at least a 97% higher likelihood of poor glycemic control in older adults. This finding suggests that the study captured unmeasured factors, and ongoing research is necessary to elucidate existing race and ethnicity–based health disparities.

Similar to previous studies,^25^ this study found that variables in the neighborhood or built environment social risk domain, especially adverse social support, were associated with poor glycemic control over time. A systematic review by Strom et al^25^ examined the association between social support and clinical and psychological outcomes, and 17 of the 37 studies that the authors reviewed reported clinical outcomes. Most studies also reported associations between higher levels of social support and improved diabetes-related clinical outcomes, including better BP and HbA_1c_ and lipid levels.^25^ Although the present study found that adverse social support was consistently associated with poor glycemic control, the association between adverse social support and poor cholesterol control was lost when depression and perceived everyday discrimination were added to the model. In addition, we found no association between neighborhood or built environment and BP control. Contrary to the findings in this study, a longitudinal study of 2280 Black adults aged 18 to 30 years reported that the neighborhood or built environment (neighborhood-level racial residential segregation) was associated with increased SBP but not DBP.^15^ Differences in methods and population sample may explain the contrast in these findings.

Consistent evidence supports the association between economic instability and adverse diabetes outcomes. A longitudinal study by Walker et al,^26^ for instance, examined the role of SDOH in glycemic control and found that, compared with psychosocial and neighborhood factors, economic stability (financial hardship) was associated with poor glycemic control over time. Although we found that economic stability (medication cost–related nonadherence) was associated with poor BP control over time, the association between economic stability (lowest-quartile income or asset) and glycemic control was lost after education access (lack of education) was added to the model. Evidence suggests that a high prevalence of adverse health behaviors among individuals with exposure to economic instability may be an underlying mechanism for this association.^26^ Chronic stress related to competing financial priorities, such as procuring food, prescribed medications, and other living expenses, is potentially associated with worse health outcomes.

The health care access social risk domain (lack of health insurance) was associated with glycemic, BP, and cholesterol control outcomes over time in partially or fully adjusted models. Evidence suggests that decreased office visits with a health care practitioner and fewer prescribed medications are associated with a lack of health insurance coverage, which could explain this finding.^5^ In addition, having health insurance is associated with increased probability of accessing diabetes care programs, including health and self-management education that could improve control of CVD risk factors and outcomes.^5^ However, research also shows that health insurance coverage alone is insufficient in closing current health gaps.^27^ For instance, a national survey of 8000 US adults that sought to examine disparities in perceived quality and affordability of care after the implementation of the Affordable Care Act found that those with lower income rated their quality of care poorly, and health insurance coverage explained only 10% to 25% of this disparity.^27^ Taken together, health care access is a complex and multidimensional concept and often includes an individual’s ability to perceive, seek, pay for, engage in, and reach medical care.^6^ Studies incorporating other dimensions of health care access are needed to confirm or refute the findings of this cohort study.

The findings highlight the need for health care practitioners to optimize the control of CVD risk factors in populations with diabetes. First, clinicians can modify health care delivery and clinical decision-making according to patient-specific social risk factors, a strategy referred to as *social risk–informed care*.^28^ Second, clinicians can leverage the patient-clinician encounter to target social risk factors in improving health outcomes, a strategy referred to as *social risk–targeted care*.^28^ Furthermore, the findings suggest social risk intervention targets may vary in older adults with diabetes, depending on which CVD risk factor they seek to control. Given that addressing upstream factors are critical to modifying individual social risk, federal or state public health policies geared toward managing the health of the community are essential. Policy makers and health systems must partner with and invest financially in their communities to improve health and health outcomes.^28^ Public health policy changes that support sustained employment opportunities and health care coverage as well as social support resources cannot be overemphasized.^29,30,31^

### Strengths and Limitations

This study has some strengths. It used a longitudinal data set and examined multiple social risk domains, which were based on the categories in the modified Kaiser Family Foundation SDOH framework.^22^

The study also has several limitations. First, social risk domains were limited by the variables available in the HRS data set. There is no consensus or guideline on social risk domains, which restricts a comparison with other studies. However, we used a well-recognized SDOH framework to identify and categorize social risk domains to increase the applicability of results. Second, this study focused on older adults with diabetes, which precludes generalization to younger populations or those without diabetes. Third, CVD risk factor measures were limited to those available in the HRS data set. We used a conservative value for TC/HDL ratio estimation, which could potentially underestimate lipid control for female individuals.^32,33^ Fourth, the study did not investigate the mechanisms underlying the associations between social risk domains and CVD risk factor control. Future studies may incorporate measures to capture the possible mechanisms of observed associations and use methods, such as mediation analysis or path analysis, to understand these pathways.

## Conclusions

This cohort study revealed that the social risk domains of neighborhood or built environment, economic stability, and health care access were associated with poor control of CVD risk factors over time. In addition, lack of health care access may be associated with decreased control of CVD risk factors over time in older adults with diabetes, and race and ethnicity and sex have implications for poor CVD risk factor control over time independent of social risk factors. Future interventions targeting specific social risk domains, including neighborhood or built environment, economic stability, and education access, may be beneficial to CVD risk factor control in older adults with diabetes.

## Notes

Supplement. **eTable.** Unadjusted Mixed Effects Logistic Regression with Social Risk Factor Domains
