# Supplementary material for: Association of Social Risk Domains With Poor Cardiovascular Risk Factor Control in US Adults With Diabetes, From 2006 to 2016
Source: JAMA Netw Open. 2022 Sep 9;5(9):e2230853. doi: 10.1001/jamanetworkopen.2022.30853 (PMC9463604; doi:10.1001/jamanetworkopen.2022.30853)
Supplement: Supplement. — eTable. Unadjusted Mixed Effects Logistic Regression with Social Risk Factor Domains [file jamanetwopen-e2230853-s001.pdf]

Supplemental Online Content

Corwin T, Ozieh MN, Garacci E, Walker RJ, Egede LE. Association of social risk domains with poor cardiovascular risk factor control in US adults with diabetes, from 2006 to 2016. *JAMA Netw Open*. 2022;5(9):e2230853. doi:10.1001/jamanetworkopen.2022.30853

eTable. Unadjusted Mixed Effects Logistic Regression with Social Risk Factor Domains

This supplemental material has been provided by the authors to give readers additional information about their work.

© 2022 Corwin T et al. *JAMA Network Open.*

eTable. Unadjusted Mixed Effects Logistic Regression with Social Risk Factor Domains[a,b]

	Blood Hemoglobin: (HbA1C) ≥ 8.0%)	Blood Pressure: SBP ≥ 140 and BP ≥ 90	Cholesterol: TC/HDL ≥ 5	CVD Risk at least 2 out of control: Yes
	OR (95% CI)	OR (95% CI)	OR (95% CI)	OR (95% CI)
**Economic Stability**				
Medication Cost Non-Adherence	1.17 (0.86 to 1.59)	1.47 (1.11 to 1.96)[a]	1.21 (0.93 to 1.57)	1.29 (0.92 to 1.82)
Difficulty Paying Bills	1.31 (0.84 to 2.04)	1.08 (0.72 to 1.61)	1.23 (0.84 to 1.80)	1.34 (0.81 to 2.23)
Financial Hardship	1.25 (0.77 to 2.02)	0.98 (0.64 to 1.52)	0.85 (0.56 to 1.27)	1.09 (0.62 to 1.88)
Lowest Quartile Income/Assets	1.38 (1.10 to 1.73)[a]	1.40 (1.14 to 1.72)[a]	1.14 (0.95 to 1.37)	1.34 (1.04 to 1.73)[a]
Employment Adversity	1.25 (0.97 to 1.63)	1.02 (0.80 to 1.30)	1.08 (0.87 to 1.33)	0.96 (0.71 to 1.29)
**Neighborhood/Built Environment**				
Food Insecurity	1.72 (1.20 to 2.47)[a]	1.57 (1.14 to 2.18)[a]	0.95 (0.70 to 1.30)	1.11 (0.73 to 1.69)
Neighborhood Physical Disorder	1.12 (0.86 to 1.46)	1.18 (0.92 to 1.52)	0.96 (0.77 to 1.19)	1.10 (0.81 to 1.49)
Lack of Neighborhood Social Cohesion	1.48 (1.14 to 1.93)[a]	0.93 (0.72 to 1.20)	1.06 (0.85 to 1.32)	1.25 (0.92 to 1.68)
Adverse Social Support	1.56 (1.26 to 1.92)[a]	1.00 (0.82 to 1.22)	1.24 (1.04 to 1.46)[a]	1.32 (1.05 to 1.67)[a]
**Education/Access**				
Lack of Education	1.62 (1.26 to 2.07)[a]	1.33 (1.08 to 1.65)[a]	1.25 (1.03 to 1.50)[a]	1.37 (1.06 to 1.78)[a]
**Health Care Access**				
Not Having Health Insurance	1.99 (1.49 to 2.66)[a]	1.86 (1.43 to 2.42)[a]	1.51 (1.20 to 1.91)[a]	2.60 (1.93 to 3.51)[a]
**Social/Community Context**				
Depression	1.61 (1.26 to 2.07)[a]	1.21 (0.96 to 1.52)	1.15 (0.94 to 1.40)	1.22 (0.92 to 1.61)
Perceived Everyday Discrimination	1.25 (1.00 to 1.55)[a]	1.13 (0.93 to 1.38)	1.21 (1.02 to 1.43)[a]	1.33 (1.05 to 1.69)[a]

[a] p-value < 0.05
[b] Each social risk factor domain is a separate model

© 2022 Corwin T et al. *JAMA Network Open.*
